# Nano-Interstice Driven Powerless Blood Plasma Extraction in a Membrane Filter Integrated Microfluidic Device

**DOI:** 10.3390/s21041366

**Published:** 2021-02-15

**Authors:** Jaehoon Kim, Junghyo Yoon, Jae-Yeong Byun, Hyunho Kim, Sewoon Han, Junghyun Kim, Jeong Hoon Lee, Han-Sang Jo, Seok Chung

**Affiliations:** 1 School of Mechanical Engineering, Korea University, Seoul 02841, Korea; kimhuks@korea.ac.kr (J.K.); navarm@naver.com (J.-Y.B.); khh8518@korea.ac.kr (H.K.); einstein.email@gmail.com (S.H.); coolkj37@gmail.com (J.K.); 2 Department of Electrical Engineering and Computer Science, Massachusetts Institute of Technology, Cambridge, MA 02142, USA; jhyoon7@mit.edu; 3 Department of Electrical Engineering, School of Electronics and Information Technology, Kwangwoon University, Seoul 01886, Korea; jhlee@kw.ac.kr; 4 Absology, Digitalempire B-dong, 383, Simin-daero, Dongan-gu, Anyang-si, Gyeonggi-do 14057, Korea; hansjo@absology.co.kr; 5 KU-KIST Graduate School of Converging Science and Technology, Korea University, Seoul 02841, Korea

**Keywords:** microfluidics, point-of-care testing, blood plasma extraction

## Abstract

Blood plasma is a source of biomarkers in blood and a simple, fast, and easy extraction method is highly required for point-of-care testing (POCT) applications. This paper proposes a membrane filter integrated microfluidic device to extract blood plasma from whole blood, without any external instrumentation. A commercially available membrane filter was integrated with a newly designed dual-cover microfluidic device to avoid leakage of the extracted plasma and remaining blood cells. Nano-interstices installed on both sides of the microfluidic channels actively draw the extracted plasma from the membrane. The developed device successfully supplied 20 μL of extracted plasma with a high extraction yield (~45%) in 16 min.

## 1. Introduction

Blood plasma is a primary source of biomarkers in various clinical diagnoses, including infectious diseases, autoimmune diseases, inflammation, and even cancers [1]. It comprises proteins, electrolytes, urea, glucose, circulating nucleic acids, bacteria, viruses, etc., and represents the physiological condition of the human body. Blood plasma accounts for approximately 55% of whole blood after removing the solid contents, i.e., red blood cells, white blood cells, and platelets. In diagnostic applications, the solid contents should be carefully removed to avoid the hindrance of biomarker signals during detection. Breakdown of red blood cell membranes, called hemolysis, causes 40–70% of sample rejection in laboratories [2]. It also disrupts the detection of microRNA levels, proteins, and metabolites [3].

Centrifugation is a representative method for blood plasma extraction. Although hand powered centrifugation methods have been developed for resource-limited point-of-care testing (POCT) applications [4,5], the extraction could depend on the user’s skill and handling. Other methods that target POCT applications suffer from various issues (Table 1). In Table 1, many extraction methods require dilution of whole blood with a high hematocrit (40–45% for healthy individuals [6]), which causes operation failure, such as clogging and low separation efficiency [7,8,9]. The dilution reduces the concentration of target analytes and, therefore, decreases sensitivity and increases reaction time for detection, which increases the chance for analyte disruption by hemolysis. It also results in low yield, defined as extracted plasma divided by the total volume of plasma. Therefore, blood plasma extraction requires minimal dilution and operating time and a large yield from a small quantity of whole blood. The dilution should be performed using extracted plasma than whole blood to avoid hemolysis and degradation in terms of POCT application.

Microfluidics have been highlighted for blood plasma separation in POCT applications due to the significant advantages of low sample and reagent volume requirement, and deliverable portability. Microfluidic blood plasma separation can be simply categorized into active or passive techniques. Active techniques utilize external force fields—such as centrifugal [4,10,11,12,13] and electric [14,15,16]—while passive techniques do not require additional instrumentation. Methods using membrane filter [17,18,19,20,21,22,23], Fahraeus effect [24], bifurcation [25], and gravitational sedimentation [26,27] are good examples of passive microfluidic passive techniques, useful for POCT applications; however, they still suffer from low yields and small extraction volume [28].

Here, a membrane filter-based microfluidic blood plasma separation device that does not require any external power is proposed. Previous membrane filter integrated microfluidic devices have a slow and less efficient plasma drawing problem from a hydrophilic membrane filter to a hydrophobic plastic device. Use of hydrophilic plastic material, such as cyclic olefin copolymer or hydrophilic treatment of the hydrophobic plastic part, increased the chance of residual blood component leakage [17]. In the developed device, a robust Nano-interstice (NI)-driven liquid filling technique was integrated to efficiently draw extracted plasma from the hydrophilic filter surface into a microfluidic channel [29,30,31,32]. A dual polystyrene cover was newly designed to achieve both goals of avoiding leakage and easy mass production. The extracted plasma was designed to fill the microfluidic channel, without any additional instrumentation only by NI-driven filling for sensing.

## 2. Materials and Methods

### 2.1. Blood Sample Preparation

Five milliliters of blood from healthy donors was collected in EDTA vacutainers (Ethylene DiamineTetraAcetic acid vacutainer, BD Corporation) to avoid coagulation. All samples were handled without dilution and were refrigerated if they were not immediately used. Samples older than 72 h were discarded.

### 2.2. Membrane Filter Evaluation

Commercially available filter membranes, Vivid Vertical Plasma Separation Membrane GR grade (Pall Corporation), were cut and prepared to sizes of 10 × 10 mm^2^, 12 × 12 mm^2^, 14 × 14 mm^2^, and 16 × 16 mm^2^. The absorbent pad was fixed on an electronic scale using tape and a membrane filter was placed on the fixed absorbent pad. Whole blood with various volumes (50, 80, and 100 μL) were applied to the membrane filter and the separated plasma was absorbed into the absorbent pad. The volume of separated plasma in the absorbent pad was measured to calculate the separation yield, i.e., the volume ratio of extracted to total plasma.

## 3. Results and Discussions

### 3.1. Fabrication of Dual-Cover Microfluidic Device

Three parts (a bottom substrate with a microchannel and two covers (one for the filter region and another for the microfluidic channel)) of the microfluidic device were injection-molded of polystyrene (PS). The bottom substrate had a filter region with a pillar array and a microfluidic channel region with NIs. Each part was assembled via a solvent-injection method under an in-house press (0.5 MPa) [29,30,31,32]. The NIs were formed via 1.5 μL of acetone injected around the channel region and dissolved outside of the microfluidic channel wall. The pressure was maintained for 35 s to ensure the formation of a closed microfluidic channel with NIs. After the microfluidic channel assembly, a commercially available filter membrane was placed on the filter region. The filter cover was then bonded using the same acetone injection method under pressure.

### 3.2. Manufacturing of Membrane Filter Integrated Microfluidic Device

**Figure 1:**
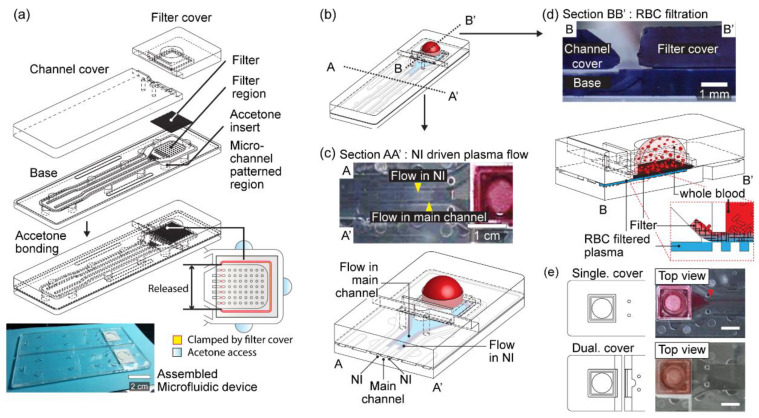
Overall illustration of the plasma separation microfluidic device with the dual cover. (**a**) Fabrication process of the device and the result of fabrication. (**b**) Application of whole blood on the device and feature of the device. (**c**) Section AA’ represents the process of plasma flow by the nano-interstice (NI)-driven flow. (**d**) Section BB’ indicates the mechanical prevention of red blood cell leakage by the dual cover system. (**e**) Whole blood application to single- and dual-cover devices. The red arrowhead indicates blood leakage. Scale bar, 4 mm.

The developed dual covered microfluidic device, one bottom substrate (base), and dual covers was presented (Figure 1). The base had two regions: a filter region with a pillar array and a microfluidic channel region. The base and channel cover were first assembled and a membrane filter was then placed on the pillar array of the base beneath the filter cover and assembled device. All parts were assembled via the solvent injection bonding method described previously (Figure 1a,b) [29,30,31,32].

The role of the pillar array under the membrane filter was to draw separated plasma from the membrane filter and transfer it to the main channel via wetting [33,34]. It has 7 × 7 pillars of 0.25 mm radius and 1.25 mm spacing. The pillars also support the wet membrane filter from touching the bottom surface and not being loose. They distribute the separated plasma in the filter region before reaching the entrance of the main channel (Figure 1c). The plasma volume that could be captured in the filter region was approximately 9.4 μL.

The NIs were installed at both sides of the main microfluidic channel, defined by the unbonded space between the channel wall and cover at C and E (Figure S1). The NI increases Young–Laplace pressure at the air–liquid interface in the sub-micron scale height of NI, which enhances wetting of the connected main channel [29]. The NI driving mechanism in microfluidic channel enables robust filling of the sample liquid even in a commercialized microfluidic device [32]. The NI-driven liquid filling powerfully draws the plasma into the main microfluidic channel. During the bonding of the base and filter cover, three sides of the membrane filter were tightly clamped but one side was released over the assembled channel cover (Figure 1d). The dual cover design, with a released membrane filter on the channel cover, successfully minimized leakage of the blood components into the main channel (Figure 1e).

### 3.3. Membrane Filter Performance for Blood Plasma Separation

**Figure 2:**
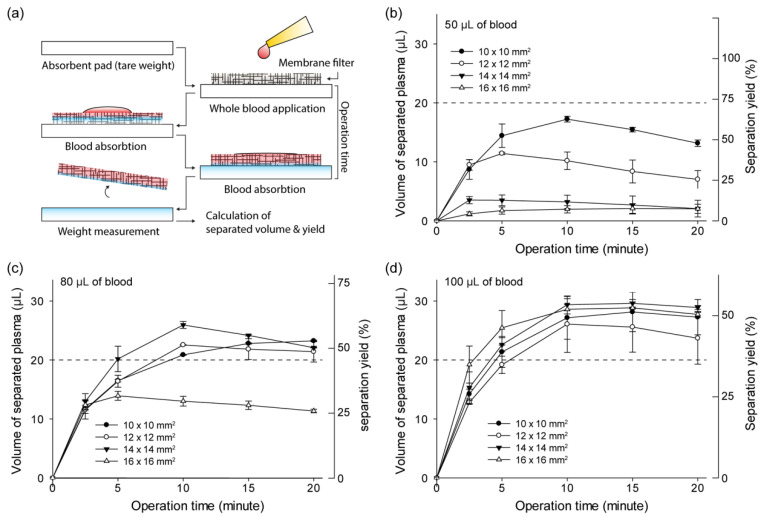
Evaluation of plasma separation performance of membrane filter according to the filter sizes and volume of whole blood. (**a**) Weight-based evaluation process using the absorbent pad. Blood plasma separation volume and yield of the membrane filter from 50 μL (**b**), 80 μL (**c**), and 100 μL (**d**) of whole blood (N = 8, error bars indicate standard deviation).

The blood plasma separation performance of the commercial membrane filter was evaluated (Figure 2). The weight of the collected plasma in the absorbent pad through the membrane filter was measured and calculated to volume. The volume of separated plasma (VoSP) and separation yield decreased as the filter size increased from $10\;\times \;10\;\;{\mathrm{mm}}^{2}$ to $16\;\times \;16\;\;{\mathrm{mm}}^{2}$ owing to the amount of residual plasma in the membrane filter. The minimum requirement of 20 μL of extracted plasma for analysis is marked on the graphs. When 80 μL or 100 μL of whole blood was applied, the volume of separated plasma reached its maximum with a filter of $14\;\times \;14\;{\mathrm{mm}}^{2}$ in 10 min of operating time. When a small amount of whole blood (50 μL and 80 μL) was applied, the separated plasma volume gradually decreased over 10 min due to evaporation. The maximum yield of the membrane filter was proven to be approximately 50%.

### 3.4. Plasma Extraction Performance in the Dual Covered Microfluidic Device

**Figure 3:**
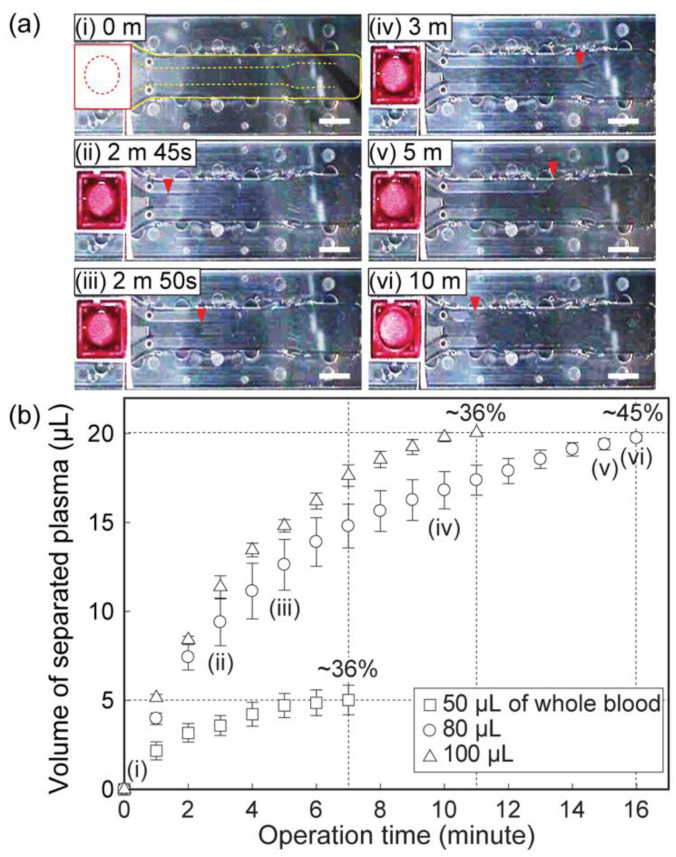
Plasma extraction in the dual cover microfluidic chip. (**a**) Images of plasma extraction in the chip depending on time. The red solid line and dotted line indicate the filter and whole blood insertion parts, respectively. The yellow solid line and dotted line represent acetone bonded edge and NI channel, respectively. Scale bars, 2 mm. (**b**) Graph of the volume of separated plasma into the microchannel.

The yield of blood plasma extraction was monitored using a CCD camera (Figure 3a). Applied whole blood, with various volumes of 50 μL, 80 μL, and 100 μL, was first vertically absorbed into the membrane filter, capturing plasma in the filter region. The separation yield of the microfluidic device was evaluated using only the volume of the plasma in the main channel, not considering plasma in the filter region. The volume of the main channel was designed to be 20 μL. The operating time was defined as the duration of the channel filling with plasma. Figure 3b shows the successful acquisition of 20 μL of extracted plasma from 80 μL and 100 μL of whole blood. The yield of 50 μL of whole blood of the developed device was approximately 36%, with 5 μL of plasma acquisition. A maximum yield of 45% was achieved in 16 min when 100 μL of whole blood was applied. Twenty microliters of plasma was easily acquired from 100 μL of whole blood in 11 min, with a 36% yield. Note that 20 μL of plasma is sufficient for various diagnostic applications [31].

### 3.5. Comparison with Previous Extraction Methods

**Figure 4:**
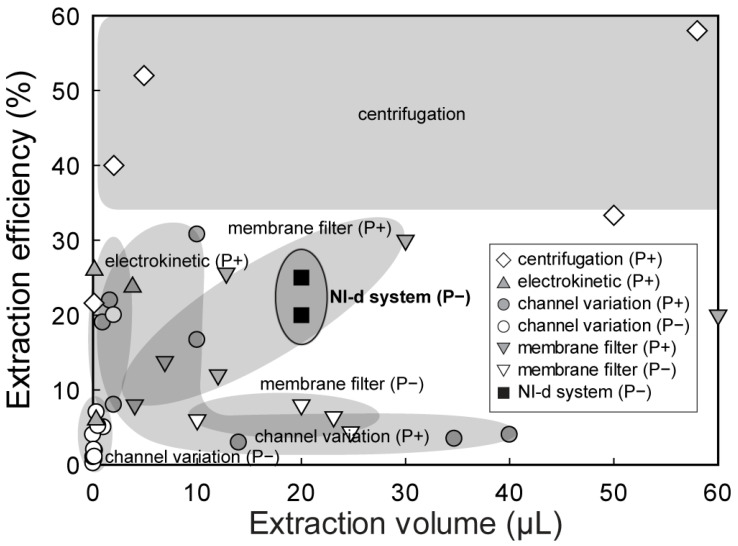
Position of plasma extraction efficiency graph. The solid rectangle represents the developed system. P+: External Power Type, P−: Powerless Type.

Plasma extraction efficiency, different from yield, was defined as the ratio of the volume of extracted plasma to that of whole blood. Figure 4 shows a graph of the extraction efficiency and extraction volume of previous plasma extraction methods. Instead of yield, extraction efficiency was adapted in the graph because some of the references did not supply hematocrit information of the whole blood samples. From the various methods, centrifugation boasts the highest (more than 30%) efficiency and a wide range of extraction volumes [4,10,11,12,13]. Generally, microfluidic methods have a wide efficiency range but very limited extraction volume due to the small scale of the channel. For example, electro-kinetic techniques show a limited extraction volume of less than 5 μL owing to their limited working flow rate [14,15,16]. Structural interruption in microfluidic channels can extract plasma via just capillary forces (P−) or with external force fields due to a pump or pressure regulator (P+). Structural interruption techniques without an external force field (P−) can only extract a very limited volume of plasma (less than 3 μL), with low efficiency of less than 10% [35,36,37,38,39,40,41,42,43,44,45], but with an external force field the extraction volume [24,26,46,47] or efficiency [25,27,48,49] are increased. Similarly, membrane filters can extract relatively large amounts of plasma without an external force field (10–20 μL) but with a very limited extraction efficiency of less than 8%, even in commercial kits [17,23]. Extraction of a large volume of plasma with relatively high efficiency (20–30%) requires additional instrumentation to apply the pressure (P+) to the blood on the membrane filter. Table 1 summarizes the previous methods used to extract plasma from whole blood. The dual cover design with a membrane filter can extract more than 20 μL of plasma, with an improved extraction efficiency of 20−25% and without any external instrumentation.

## 4. Conclusions

In conclusion, we developed an optimized dual-cover microfluidic chip for plasma separation without channel clogging and red blood cell leakage using a membrane filter, two cover designs, and NIs. The developed system successfully satisfied four requirements for POCT plasma separation. Our system accomplished (1) the use of whole blood, (2) high extraction yields of 36–45% within 100 μL, (3) 16 min of operating time, and (4) powerless operation. The developed chip exhibited 20–25% plasma extraction efficiency, which significantly improved upon previous powerless membrane filter techniques. However, operation time is still longer than other active plasma extraction methods, which could be reduced by an additional optimization of filter membrane, channel structure, and characteristics of channel surface. Integration with small and easy instrumentation, i.e., hand-powered pump [50] could be additional solution to reduce the required operation time. Possible absorption of target proteins to the filter membrane could limit highly sensitive detection. We expect that our system could be applied for the detection of diseases with only ~20 µL plasma, for analysis via integration of immunoassay technology into the downstream portion of the straight channel. The developed device to be improved as an on-chip immunoassay platform using whole blood, by integrating our previous published protocols using diluted plasma [31,32]. Despite the successful development of a powerless plasma separation platform, we need to investigate the number of blood cells in the extracted plasma and to validate the recovery rate of proteins, metabolites, and nucleic acids for diagnostic applications in future studies.

## Figures and Tables

**Table 1:** Efficiency map of the channel variation methods. PD-10 and PD-25 indicate product number of PlasmaDrop Kits for Free Liquid Plasma from MDI Membrane Technologies INC. WB: whole blood, HCT: hematocrit, N.R: No reference, D: dilution. Plasma efficiency (%): volume percent between separated plasma and input blood.

Filtration Method	Type	WB Volume (μL)	HCT (%)	Extraction			REF
				Volume (μL)	Efficiency (%)	Time (s)	
Centrifugal (Active)	External power	5	44	2	40	20	10
		100	N.R	58	58	480	4
		9.4	48	4.89	52	200	11
		150	N.R	50	33.33	180	12
		0.5	6(D)	0.108	21.6	1	13
Electrokinetic (Active)	External power	5	10(D)	0.3	6	600	14
		0.5	N.R	0.13	26	N.R	15
		16	40	3.8	23.75	180	16
Channel variation (Passive)	External power	60	N.R(D)	10	16.67	360	48
		475	20(D)	38	8	3600	46
		5	25(D)	0.95	19	1800	25
		1000	30(D)	34.7	3.47	3600	24
		1000	53	40	4	300	47
		7.5	N.R(D)	1.645	21.93	300	49
		32.5	50(D)	10	30.77	1560	27
		25	N.R	2	8	312.5	26
	Powerless	20	N.R	0.15	0.75	180	35
		10	50	0.02	0.2	N.R	36
		20	N.R	1	5	120	37
		0.2	N.R	0.008	4	2	38
		2	N.R	0.02	1	25	39
		10	N.R	0.19	1.88	600	40
		5	43	0.35	7	110	41
		10	N.R	0.51	5.17	490	42
		10	N.R	2	20	900	43
		5	45	0.1	2	300	44
		15	N.R	0.16	1.07	N.R	45
Membrane filter (Passive)	External power	100	N.R	12	12	420	18
		50	11(D)	12.8	25.6	20	19
		50	43	4	8	20	19
		100	45	30	30	420	20
		300	N.R	60	20	300	21
		50	N.R	6.9	13.8	600	22
	Powerless	225	43	20	8	600	17
		150	N.R	10	6.67	300	PD-10
		450	N.R	25	5.55	300	PD-25
		340	N.R(D)	23.5	6.9	1200	23

## Data Availability

Not applicable.

## Supplementary Materials

The following are available online at https://www.mdpi.com/1424-8220/21/4/1366/s1, Figure S1: Measured height of the device after acetone bonding (n = 3, Error bars indicate standard deviation) (ST4080-OSP, K-MAC, Daejeon, Korea).

## References

[1] Stern E., Vacic A., Rajan N.K., Criscione J.M., Park J., Ilic B.R., Mooney D.J., Reed M.A., Fahmy T.M. (2010). Label-Free biomarker detection from whole blood. Nat. Nanotechnol..

[2] Mielczarek W.S., Obaje E.A., Bachmann T.T., Kersaudy-Kerhoas M. (2016). Microfluidic blood plasma separation for medical diagnostics: Is it worth it?. Lab Chip.

[3] Asirvatham J.R., Moses V., Bjornson L. (2013). Errors in potassium measurement: A laboratory perspective for the clinician. N. Am. J. Med. Sci..

[4] Wong A.P., Gupta M., Shevkoplyas S.S., Whitesides G.M. (2008). Egg beater as centrifuge: Isolating human blood plasma from whole blood in resource-poor settings. Lab Chip.

[5] Liu C.H., Chen C.A., Chen S.J., Tsai T.T., Chu C.C., Chang C.C., Chen C.F. (2018). Blood plasma separation using a fidget-spinner. Anal. Chem..

[6] Kim S., Ma Y., Agrawal P., Attinger D. (2016). How important is it to consider target properties and hematocrit in bloodstain pattern analysis?. Forensic Sci. Int..

[7] Jäggi R.D., Sandoz R., Effenhauser C.S. (2007). Microfluidic depletion of red blood cells from whole blood in high-aspect-ratio microchannels. Microfluid. Nanofluidics..

[8] Holmes D., Whyte G., Bailey J., Vergara-Irigaray N., Ekpenyong A., Guck J., Duke T. (2014). Separation of blood cells with differing deformability using deterministic lateral displacement. Interface Focus.

[9] Tachi T., Kaji N., Tokeshi M., Baba Y. (2009). Simultaneous separation, metering, and dilution of plasma from human whole blood in a microfluidic system. Anal. Chem..

[10] Haeberle S., Brenner T., Zengerle R., Ducrée J. (2006). Centrifugal extraction of plasma from whole blood on a rotating disk. Lab Chip.

[11] Li T., Zhang L., Leung K.M., Yang J. (2010). Out-Of-Plane microvalves for whole blood separation on lab-on-a-CD. J. Micromech. Microeng..

[12] Lee B.S., Lee J.-N., Park J.-M., Lee J.-G., Kim S., Cho Y.-K., Ko C. (2009). A fully automated immunoassay from whole blood on a disc. Lab Chip.

[13] Zhang J., Guo Q., Liu M., Yang J. (2008). A lab-on-CD prototype for high-speed blood separation. J. Micromech. Microeng..

[14] Nakashima Y., Hata S., Yasuda T. (2010). Blood plasma separation and extraction from a minute amount of blood using dielectrophoretic and capillary forces. Sens. Actuators B Chem..

[15] Jiang H., Weng X., Chon C.H., Wu X., Li D. (2011). A microfluidic chip for blood plasma separation using electro-osmotic flow control. J. Micromech. Microeng..

[16] Doria A., Patel M., Lee A.P. Rapid blood plasma separation with air-liquid cavity acoustic transducers. Proceedings of the 15th International Conference on Miniaturized Systems for Chemistry and Life Science 2011, MicroTAS 2011.

[17] Thorslund S., Klett O., Nikolajeff F., Markides K., Bergquist J. (2006). A hybrid poly(dimethylsiloxane) microsystem for on-chip whole blood filtration optimized for steroid screening. Biomed. Microdevices.

[18] Homsy A., van der Wal P.D., Doll W., Schaller R., Korsatko S., Ratzer M., Ellmerer M., Pieber T.R., Nicol A., de rooij N.F. (2012). Development and validation of a low cost blood filtration element separating plasma from undiluted whole blood. Biomicrofluidics.

[19] Kobayashi T., Konishi S. (2012). Microfluidic chip with serially connected filters for improvement of collection efficiency in blood plasma separation. Sens. Actuators B Chem..

[20] Im S.B., Kim S.C., Shim J.S. (2016). A smart pipette for equipment-free separation and delivery of plasma for on-site whole blood analysis. Anal. Bioanal. Chem..

[21] Su X., Zhang S., Ge S., Chen M., Zhang J., Zhang J., Xia N. (2018). A low cost, membranes based serum separator modular. Biomicrofluidics.

[22] Shimizu H., Kumagai M., Mori E., Mawatari K., Kitamori T. (2016). Whole blood analysis using microfluidic plasma separation and enzyme-linked immunosorbent assay devices. Anal. Methods.

[23] Wang S.Q., Sarenac D., Chen M.H., Huang S.-H., Giguel F.F., Kuritzkes D.R., Demirci U. (2012). Simple filter microchip for rapid separation of plasma and viruses from whole blood. Int. J. Nanomed..

[24] Rodríguez-Villarreal A.I., Arundell M., Carmona M., Samitier J. (2010). High flow rate microfluidic device for blood plasma separation using a range of temperatures. Lab. Chip.

[25] Yang S., Ündar A., Zahn J.D. (2006). A microfluidic device for continuous, real time blood plasma separation. Lab Chip.

[26] Forchelet D., Beguin S., Sajić T., Bararpour N., Pataky Z., Frias M., Grabherr S., Augsburger M., Liu Y., Charnley M. (2018). Separation of blood microsamples by exploiting sedimentation at the microscale. Sci. Rep..

[27] Xie Y., Chen D., Lin S., Wang Z., Cui D. (2016). A robust and easily integrated plasma separation chip using gravitational sedimentation of blood cells filling-in high-aspect-ratio weir structure. RSC Adv..

[28] Tripathi S., Varun Kumar Y.V.B., Prabhakar A., Joshi S.S., Agrawal A. (2015). Passive blood plasma separation at the microscale: A review of design principles and microdevices. J. Micromech. Microeng..

[29] Chung S., Yun H., Kamm R.D. (2009). Nanointerstice-Driven microflow. Small.

[30] Kim J., Han S., Yoon J., Lee E., Lim D.W., Won J., Byun J.-Y., Chung S. (2015). Nanointerstice-Driven microflow patterns in physical interrupts. Microfluid. Nanofluid..

[31] Yoon J., Lee E., Kim J., Han S., Chung S. (2017). Generation of digitized microfluidic filling flow by vent control. Biosens. Bioelectron..

[32] Kim J., Hong K., Kim H., Seo J., Jeong J., Bae P.K., Shin Y.B., Lee J.H., Oh H.J., Chung S. (2020). Microfluidic immunoassay for point-of-care testing using simple fluid vent control. Sens. Actuators B Chem..

[33] Zimmermann M., Schmid H., Hunziker P., Delamarche E. (2007). Capillary pumps for autonomous capillary systems. Lab Chip.

[34] Olanrewaju A., Beaugrand M., Yafia M., Juncker D. (2018). Capillary microfluidics in microchannels: From microfluidic networks to capillaric circuits. Lab Chip.

[35] Sakamoto H., Hatsuda R., Miyamura K., Sugiyama S. (2012). Plasma separation PMMA device driven by capillary force controlling surface wettability. Micro Nano Lett..

[36] Kim Y.C., Kim S.H., Kim D., Park S.J., Park J.K. (2010). Plasma extraction in a capillary-driven microfluidic device using surfactant-added poly(dimethylsiloxane). Sens. Actuators B Chem..

[37] Khumpuang S., Tanaka T., Aita F., Meng Z., Ooe K., Ikeda M., Omori Y., Miyamura K., Yonezawa H., Matsumoto K. Blood plasma separation device using capillary phenomenon. Proceedings of the TRANSDUCERS EUROSENSORS ’07—4th International Conference Solid-State Sensors, Actuators and Microsystems.

[38] Kim D., Yun J.Y., Park S.J., Lee S.S. (2009). Effect of microstructure on blood cell clogging in blood separators based on capillary action. Microsyst. Technol..

[39] Zhan Y.H., Kuo J.N. Dimensions and capillary effects of microfluidic channel for blood plasma separation. Proceedings of the 7th IEEE International Conference on Nano/Micro Engineered and Molecular Systems, NEMS 2012.

[40] Li C., Liu C., Xu Z., Li J. (2012). Extraction of plasma from whole blood using a deposited microbead plug (DMBP) in a capillary-driven microfluidic device. Biomed. Microdevices.

[41] Li C., Liu C., Xu Z., Li J. (2012). A power-free deposited microbead plug-based microfluidic chip for whole-blood immunoassay. Microfluid. Nanofluid..

[42] Shim J.S., Ahn C.H. (2012). An on-chip whole blood/plasma separator using hetero-packed beads at the inlet of a microchannel. Lab Chip.

[43] Maria M.S., Rakesh P.E., Chandra T.S., Sen A.K. (2017). Capillary flow-driven microfluidic device with wettability gradient and sedimentation effects for blood plasma separation. Sci. Rep..

[44] Madadi H., Casals-Terré J., Mohammadi M. (2015). Self-Driven filter-based blood plasma separator microfluidic chip for point-of-care testing. Biofabrication.

[45] Park S., Shabani R., Schumacher M., Kim Y.-S., Bae Y.M., Lee K.-H., Cho H.J. (2016). On-Chip whole blood plasma separator based on microfiltration, sedimentation and wetting contrast. Microsyst. Technol..

[46] VanDelinder V., Groisman A. (2006). Separation of plasma from whole human blood in a continuous cross-flow in a molded microfluidic device. Anal. Chem..

[47] Kim B., Choi S. (2016). Smart pipette and microfluidic pipette tip for blood plasma separation. Small.

[48] Kang T.G., Ji H.M., Zhang G.J., Agarwal A., Chen Y. Back-To-Back integrated nanowire biosensor with microfiltration device for application to the cardiac biomarker detection from blood sample. Proceedings of the 14th International Conference on Miniaturized Systems for Chemistry and Life Sciiences, MicroTAS 2010.

[49] Kim P., Ong E.H., Li K.H.H., Yoon Y.J., Ng S.H.G., Puttachat K. (2016). Low-Cost, disposable microfluidics device for blood plasma extraction using continuously alternating paramagnetic and diamagnetic capture modes. Biomicrofluidics.

[50] Jalal U.M., Jin G.J., Shim J.S. (2017). Paper-Plastic hybrid microfluidic device for smartphone-based colorimetric analysis of urine. Anal. Chem..

