# Supplementary material for: Nano-Interstice Driven Powerless Blood Plasma Extraction in a Membrane Filter Integrated Microfluidic Device
Source: Sensors (Basel). 2021 Feb 15;21(4):1366. doi: 10.3390/s21041366 (PMC7919272; doi:10.3390/s21041366)
Supplement: Supplementary file 1 [file sensors-21-01366-s001.pdf]

Supporting material

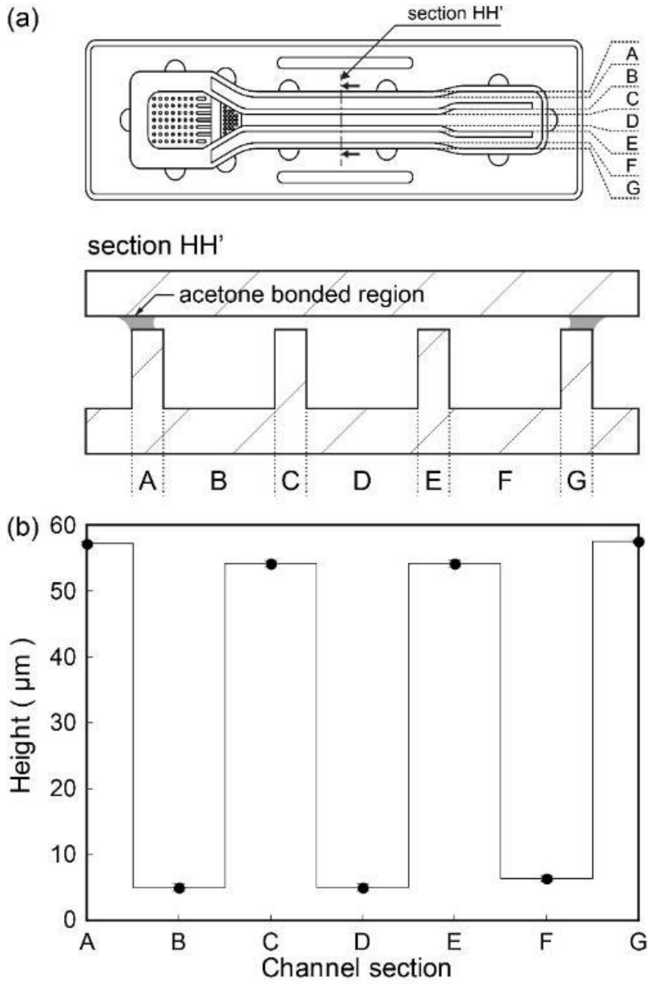

Figure 1. (**a**) Illustration of the plasma separation microfluidic device. The heights were measured at 7 points (A, B, C, D, E, F, and G) from section H-H'. (**b**) Measured height of the device after acetone bonding (n = 3, Error bars indicate standard deviation) (ST4080-OSP, K-MAC, Daejeon, Korea).
